# Macrophage migration inhibitory factor (MIF) modulates trophic signaling through interaction with serine protease HTRA1

**DOI:** 10.1007/s00018-017-2592-z

**Published:** 2017-07-19

**Authors:** Åsa Fex Svenningsen, Svenja Löring, Anna Lahn Sørensen, Ha Uyen Buu Huynh, Simone Hjæresen, Nellie Martin, Jesper Bonnet Moeller, Maria Louise Elkjær, Uffe Holmskov, Zsolt Illes, Malin Andersson, Solveig Beck Nielsen, Eirikur Benedikz

**Affiliations:** 1 Department of Molecular Medicine-Neurobiology Research, University of Southern Denmark, J.B. Winslows Vej 21.1, 5000 Odense, Denmark; 2 Tytgat Institute for Liver and Intestinal Research, Academic Medical Center, Meibergdreef 69-71, 1105 BK Amsterdam, The Netherlands; 3 Department of Neurology, Odense University Hospital, University of Southern Denmark, Sdr. Boulevard 29, 5000 Odense C, Denmark; 4 Department of Molecular Medicine-Cancer and Inflammation, University of Southern Denmark, J.B. Winslows Vej 21.1, 5000 Odense, Denmark; 5 Weill Cornell Medicine, Cornell University, 413 East 69th Street, New York, 10021 USA; 6 Department of Pharmaceutical Biosciences, Uppsala University, Box 59, 751 24 Uppsala, Sweden; 7 Faculty of Health, University College Zealand, Parkvej 190, 4700 Næstved, Denmark

**Keywords:** MIF, HTRA1, Protein interaction, Yeast-2-hybrid

## Abstract

Macrophage migration inhibitory factor (MIF), a small conserved protein, is abundant in the immune- and central nervous system (CNS). MIF has several receptors and binding partners that can modulate its action on a cellular level. It is upregulated in neurodegenerative diseases and cancer although its function is far from clear. Here, we report the finding of a new binding partner to MIF, the serine protease HTRA1. This enzyme cleaves several growth factors, extracellular matrix molecules and is implicated in some of the same diseases as MIF. We show that the function of the binding between MIF and HTRA1 is to inhibit the proteolytic activity of HTRA1, modulating the availability of molecules that can change cell growth and differentiation. MIF is therefore the first endogenous inhibitor ever found for HTRA1. It was found that both molecules were present in astrocytes and that the functional binding has the ability to modulate astrocytic activities important in development and disease of the CNS.

**Electronic supplementary material:**

The online version of this article (doi:10.1007/s00018-017-2592-z) contains supplementary material, which is available to authorized users.

## Introduction

*Macrophage migration inhibitory factor*, MIF, is a highly conserved 12.5-kDa cytokine-like protein that exhibits a unique combination of hormone-, cytokine-, and enzyme-like properties. It operates via intra- and extracellular protein–protein interactions as well as via CD74/CXCR2/CXCR4/CXCR7 receptor-mediated pathways regulating immune and inflammatory responses, cell growth, migration and apoptosis [1–7]. MIF was originally studied in the immune system where its expression is high, but MIF is, in fact, expressed in many different tissues and cells [8]. In the endocrine system, for example in the healthy pancreas, MIF is secreted together with insulin. Here MIF acts as an autocrine factor to stimulate insulin release [9]. It also binds to insulin and promotes its stability [10]. During development of the central nervous system (CNS), MIF acts as a neurotrophic growth factor and is involved in stem cell proliferation and neurogenesis as well as inner ear development [4–6, 11, 12]. Deletion of MIF in the mouse CNS results in increased anxiety and depression-like behaviors, as well as impaired hippocampus-dependent memory, likely connected to its influence on stem cells [4]. MIF mediates antidepressant actions in voluntary exercise and has the ability to increase serotonin secretion in the brain [13]. Increased MIF levels also have neuroprotective properties and are implicated in wound healing after spinal cord injury and peripheral nerve regeneration [14, 15].

An upregulation of MIF has been reported to be detrimental in several neurodegenerative diseases such as Alzheimer’s disease (AD), mild cognitive impairment, Parkinson´s disease and multiple sclerosis (MS) [16–20]. In AD, MIF has been isolated in association with the β-amyloid peptide, the main constituent of AD plaques and in vitro, β-amyloid induced toxicity could be reversed by the small molecule inhibitor of MIF; ISO-1 [21, 22]. These data together with the fact that CD74, a common receptor for MIF, is upregulated in neurofibrillary tangles in AD, suggest that MIF may be involved in the degenerative process [23].

Recent clinical studies have identified MIF as a marker of clinical worsening in MS patients [22, 24, 25]. It inhibits the action of glucocorticoids that are used as a treatment to shorten MS relapses [26]. MIF also promotes disease progression in an animal model of MS, experimental autoimmune encephalomyelitis (EAE) likely through its ability to skew the CNS inflammatory milieu [27]. It has been shown that MS patients have a higher expression of the MIF receptor CD74 on monocytes, which may make this cell type more reactive, increasing the activity in signaling pathways necessary for MAPK activation and cell motility [28, 29].

While the role of MIF in the immune system has been thoroughly studied, less is known about its function in the nervous system. To further investigate the role of MIF in the CNS, we screened a brain cDNA library for new binding partners to MIF and identified the serine protease HTRA1 as an MIF interacting protein.

HTRA1 is a highly conserved 51-kDa protein [30, 31]. It is expressed in several tissues including the brain, where it is present in most cells [32–36] The majority of the HTRA1 is secreted into the extracellular space while the remaining fraction localizes to the cytoplasm attached to microtubules [37, 38]. In the extracellular space, HTRA1 acts as an enzyme that digests a number of proteins and extracellular matrix molecules [39–43]. Intracellularly, it is also been implicated as a tumor suppressor and in cell proliferation and migration [35, 36, 40, 43, 44]. HTRA1 has been suggested to play a role in several diseases such as macular degeneration, arthritis and CARASIL [39, 45–49]. In Alzheimer’s disease HTRA1 has been implicated in the disassembly and degradation of tau and Aβ fibrils [50].

The function of the interaction was first investigated biochemically and then studied in vitro in astrocytes that express both MIF and HTRA1. In these two systems, the enzymatic activity of HTRA1 was inhibited by MIF, and consequently HTRA1-mediated degradation of growth factors was also blocked.

## Methods

Full-length human MIF and the two truncated forms were PCR amplified from MGC clone 61527 (IMAGE: 6047427, Source BioScience). The PCR fragments were purified and cloned, directly in-frame with the Gal4 DNA-BD, by In-Fusion technique (Clontech, #639690) into the pGBKT7 vector (Clontech) pre-opened with restriction enzymes EcoRI (Invitrogen, #15202-013) and BamHI (Invitrogen, #15201-023). The construct was transformed into TOP10 bacteria (Invitrogen) and grown at 37 °C ON. Colonies were selected, analyzed and sequenced using a T7 primer.

### Transformation of competent yeast cells with plasmid DNA

The Yeastmaker Yeast Transformation System 2 (Clontech, PT1172-1) was used in all transformations. Competent Y2HGold or Y187 cells (Clontech) were prepared and the vectors introduced into the yeast cells using an adapted lithium acetate (LiAc)/single-stranded DNA/polyethylene glycerol (PEG) method (Clontech). Denatured carrier DNA (50 µg, Clontech #630440) and 100 ng vector DNA was added to 50 μL competent yeast cells together with 500 μL PEG/LiAC. After 30 min incubation at 30 °C, 20 µL DMSO was added and the cells were heat-shocked at 42 °C for 15 min. The cells were spun down at high speed for 15 s, 1 mL YPD plus medium (Clontech) was added and the cells were incubated with shaking at 125 rpm at 30 °C for 30 min. After incubation, the cells were centrifuged at 115*g* for 5 min and resuspended in 1 mL 0.9% NaCl. The cell suspension was spread onto selective agar plates and grown at 30 °C for 3–5 days.

### Yeast two-hybrid screenings

The Matchmaker Gold Yeast Two-Hybrid (Y2H) system (Clontech, #630489) was used in accordance with manufacturer’s protocol to identify proteins interacting with MIF. A pre-transformed human fetal brain cDNA library (Clontech, #630469) in Y187 cells was used for the screen. The Y187 cells were mated with the haploid MATα reporter strain, Y2HGold, expressing MIF. Prior to all screenings, every construct was tested for auto-activation, toxicity and protein expression according to the manufacture’s protocol. Positive protein-interacting colonies were re-streaked several times before isolating colonies for analysis. Vector DNA was isolated from the selected colonies and interacting proteins identified by DNA sequencing.

### Co-transformation

Interacting proteins retrieved from Y2H screenings were verified by co-transformation experiments. Coding regions of interacting proteins were expressed in pGADT7 and transformed into Y2HGold expressing pGBKT MIF. Co-transformed Y2HGold were grown on selective agar plates for 3–5 days at 30 °C according to the manufacturer’s protocol.

### Software analysis of the MIF–HTRA1 interaction

To determine hypothetical MIF–HTRA1 binding sites, the program Patchdock (Beta 1.3 Version) was used to identify molecular docking. The protein data bank (PDB) files for MIF (1ca7) and HTRA1 (2joa and 3num) were used for modeling. The Patchdock results were refined using the FireDock software [51, 52].

### Microscale thermophoresis

Recombinant human HTRA1 (R&D Systems, 2916-SE) was labeled using the Monolith NT™Protein Labeling Kit RED-NHS (NanoTemper Technologies). Labeling was performed according to the manufacturers instructions in coupling buffer (50 mM HEPES, 700 mM NaCl, pH 8.2), applying a concentration of 5.5 µM HTRA1 and a molar dye to protein ratio of 5:1. Removal of unreacted dye was performed with the supplied columns equilibrated in assay buffer (50 mM Tris, 0.05% Tween-20, pH 8.0).

Labeled HTRA1 as well as the unlabeled MIF (Pepro-Tech, 300-69) were diluted in the assay buffer. The concentration of HTRA1 was kept constant at 65 nM throughout the measurements, whereas MIF was titrated in 2:1 dilutions, ranging from 2.28 nM to 1 µM. For the measurements, each MIF dilution was incubated with labeled HTRA1 for 20 min at RT before being loaded into hydrophilic glass capillaries (NanoTemper Technologies). Measurements were performed using a Monolith NT.115 instrument (NanoTemper Technologies) at an ambient temperature of 25 °C with 5 s/30 s/5 s laser off/on/off times, respectively. Instrument parameters were adjusted to 40% LED power and 80% MST power. Data from three independently pipetted experiments were analyzed using the signal from Thermophoresis + T-jump.

### Animals

C57BL/6J mice were purchased from Taconic (Denmark). The mice were kept on a 12-h dark–light cycle with food and water ad libitum. The study was approved by the regional ethics committee for research animals (Odense, Denmark). The mice pups (P1–P5) were decapitated using sharp scissors.

### Isolation of primary astrocytes

For experiments involving astrocytes, brains of P1–P5 mice were mechanically dissociated and the obtained cells cultivated in DMEM supplemented with 10% fetal bovine serum and penicillin–streptomycin for a minimum of 10 days. The protocol of de Vellis and Cole was followed for purification of astrocytes [53]. In short, the flasks containing mixed glial cultures were placed on a rotary shaker at 37 °C, 200 rpm, first for 4 h to remove microglia. The medium with microglia was discarded, and new medium was added and the flasks shaken for additional 12–18 h under same conditions. The medium was then collected and centrifuged at 1000 rpm, The flasks were washed with pre- warmed medium once and then medium containing Dulbecco’s Modified Eagle Medium (DMEM) with glutamax containing 10% fetal bovine serum (FBS) and 1× penicillin and streptomycin (Gibco) was added to the astrocytes that adhered to the bottom. Purified astrocytes were removed using trypsin and re-plated in Permanox 4 well Chamber slides (Gibco) for further processing.

### Transfection of HEK 293 cells with HTRA1

Full-length HTRA1 was cloned into the pcDNA3 mammalian expression vector kindly provided by Prof. Alfonso Baldi. HEK293 cells at a confluence of 70% were transfected with the plasmid using lipofectamine LTX transfection reagent (Invitrogen). Stable transfection was ensured by the addition of 500 µg/mL geneticin to the culture medium for several weeks.

For assays using medium of HTRA1-transfected HEK293 cells, the cells were grown until reaching a confluence of approximately 90%. The medium was changed to serum-free DMEM in a volume just covering the surface of the flask. The cultures were then incubated for 26–30 h, and the enriched medium collected, centrifuged at 1200 rpm, 5 min, and used for cleavage and proliferation assays. Medium from untransfected cell was used as a control. For all presented cleavage assays, the aliquots from the same collected batch of medium were used to ensure a constant amount of HTRA1.

### Activity assay

For the activity assay with HTRA1, substrates were used in different concentrations. β-casein (1 µg) was used as positive control, while FGF8, 0.5 µg (Pepro-Tech, 100-25), and both FGF17 (Pepro-Tech, 100-27) and FGF18 (Pepro-Tech, 100-28) were investigated as possible substrates using 0.75 µg in the assay.

To obtain active HTRA1, medium was collected from the transfected HEK293 cells. The amount of HTRA1-containing medium optimal for cleavage was first investigated as the lowest concentration cleaving 1 µg β-casein. Through testing, the lowest amount leading to visible cleavage of the substrate was found to be 4 µL, this amount was used for further analysis; medium from untransfected HEK cells was used as control (see Supplementary figure 1). To determine the actual concentration of the HTRA1 from the medium, western blots were made with the media containing HTRA1 and several concentrations of recombinant HTRA1 (R&D systems (R&D Systems, 2916-SE) 1–200 ng. The concentration of HTRA1 in the media was determined to be between 10 and 50 ng/µL.

Medium from untransfected HEK293 cells served as control and was used in the same amounts as the HTRA1 containing medium (see Supplementary figure 1). The samples were prepared in a total volume of 20 µL in assay buffer (50 mM Tris–HCl, pH 8.0). For the examination of MIF’s inhibitory effect on HTRA1, MIF (Pepro-Tech, 300-69) ranging from 0.001–5 µg was added to the lowest volume of HTRA1 cleaving β-casein, in a total volume of 20 µL in assay buffer. These proteins were incubated for 10 min before addition of 1 µg of β-casein to the samples. The samples were incubated for 2 h at 37 °C. The lowest concentration of MIF able to inhibit the enzymatic activity of HTRA1 was determined to be 1 µg and this was used for further activity studies with β-casein (see Supplementary figure 1C).

When using FGF8 and FGF18 as substrates, 12.5 µL of HTRA1-containing medium and correspondingly 3 µg MIF were used. For FGF17 and FGF18 samples were incubated overnight at 37 °C (see Supplementary figure 1).

After incubation, the samples were separated on a 15% SDS polyacrylamide gel and stained using SimplyBlue™ SafeStain (Invitrogen) according to the manufacture’s protocol. Destaining was performed overnight in ddH_2_O supplemented with 20% NaCl.

### Analysis of astrocyte migration

To investigate migration a protocol for the scratch test/wound healing was used [54, 55]. In short, Permanox 4 well Chamber slides (Gibco), were used for the procedure. In the bottom of the slides, five vertical reference lines were made with a scalpel. Primary astrocytes, 60,000 cells/mL were then seeded into the poly-D lysine coated dishes. The cells were grown for 24 h—16 of these in FBS-free medium to inhibit cell proliferation during the assay until 80% confluent. A horizontal scratch was made with a 10-µL pipette tip, and the medium was carefully changed to pre-warmed DMEM [56, 57]. Pictures were taken at *t*_0_, and the test substances were added. These included 25 ng/mL FGF8, collected HTRA1-containing medium in the ratio to FGF8 determined in the activity assay (0.12 µL), and 50 ng/mL MIF. In assays where MIF and HTRA1 were added in combination with the cells, they were pre-incubated for at least 5 min before the addition. After addition of the test substances, the cells were incubated for an additional 24 h. Pictures taken after this time were designated *t*_24_. The pictures were taken at the intersections of each reference line with the scratch, thereby ten pictures were taken from each well and it was ensured that *t*_0_ and *t*_24_ were captured at the same positions within the wells.

### Immunocytochemistry

The cells were fixed in 4% PFA for 10 min and washed three times in washing buffer. After washing three times in washing buffer (PBS + 0.25% Triton X-100 + 025% BSA), the slides containing astrocytes were blocked in blocking solution (PBS + 0.25% Triton X-100) for 30 min at room temperature. Incubation with the corresponding antibodies diluted in blocking solution was done overnight at 4 °C. The following primary antibodies were used: primary polyclonal rabbit anti-MIF antibody (1:200; Abcam ab7207), primary polyclonal rabbit HTRA1 (1:200, A kind gift from Michael Ehrmann at Universität Duisburg-Essen, Essen, Germany), primary polyclonal chicken anti-GFAP (1:1.000; Abcam, ab4674), polyclonal rabbit anti-FGF8 (1:200, Abcam, ab81384) and polyclonal mouse anti-FGF18 (1:200, Abcam, ab169615). Incubation with the secondary antibodies was performed in the dark at room temperature for 1 h. The secondary antibodies were as following: Alexa Fluor-488 goat anti-rabbit IgG (1:400; Invitrogen, 828814), Rhodamine Red-X goat anti-chicken (1:400; Jackson Immuno Research, 93951) and Rhodamine Red-X goat anti-mouse (1:400; Jackson Immuno Research, 94085). The slides were washed in PBS and stained with DAPI for 20 min before mounting. For all staining procedures, negative control antibodies were performed.

### Statistics

For the analysis of band intensities, pictures were taken and analyzed using the program MultiGauge (Fujifilm) and the Quick Guide for Quantitative Analysis [Quant (Fujifilm)]. The (Q-B)/pixel2 values of bands were standardized to the corresponding control sample. The data were compared statistically using one-way ANOVA in combination with the Bonferroni’s correction with a significance threshold at *p* < 0.05.

The scratch area (area without cells) was calculated for each picture using ImageJ. The *t*_24_ value was subtracted from the corresponding *t*_0_ value, and designated as the reduction in scratch area given in mm^2^. An average was calculated from all ten pictures of each well (10–18 wells per condition, 6 different experiments). With the program GraphPad Grubb’s test, the values were analyzed for outliers, which were then removed manually. Statistical analysis was done using the GraphPad and one-way ANOVA in combination with the Bonferroni’s correction with a significance threshold at *p* < 0.05. BrdU was added during the 24 h. The cultures were fixed and treated as described below. Immunocytochemistry of the cultures was performed using BrdU, to investigate proliferation. This investigation showed no significant difference between control and treated cells (data not shown).

## Results

### Finding new binding partners to MIF

**Fig. 1:**
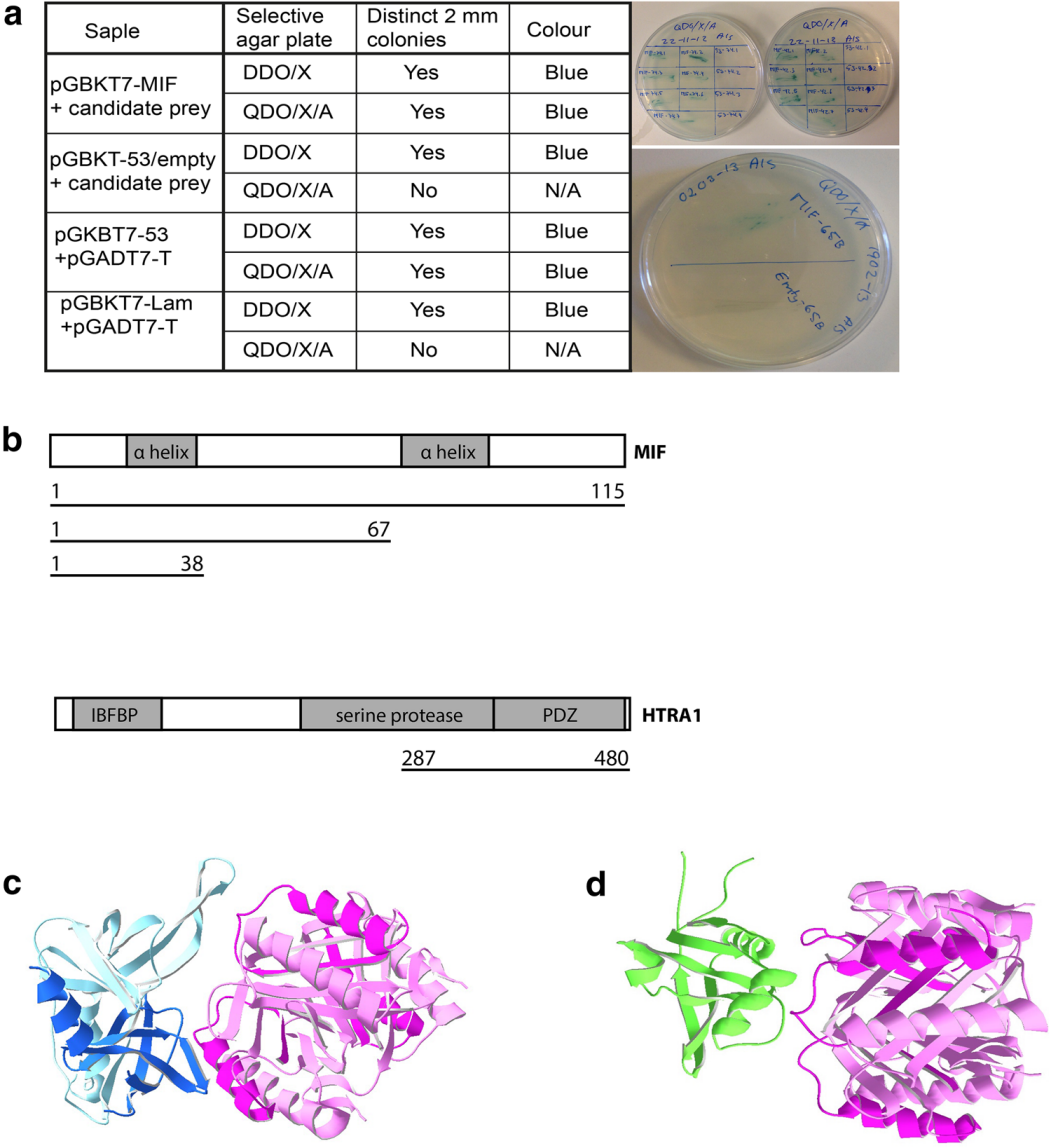
Identification of protein–protein binding: to identify proteins that bind to full-length MIF, a yeast two-hybrid screen was performed using a human fetal brain cDNA library. In this screen HTRA1 was identified as a binding partner to MIF. **a** Interacting proteins from Y2H screenings were verified by co-transformation experiments. Coding regions of interacting proteins were expressed in pGADT7 and transformed into Y2HGold expressing pGBKT MIF. Co-transformed Y2HGold were grown on selective agar plates for 3–5 days at 30 °C according to the manufacturer’s protocol. The results after co-transformation of Y2HGold with pGBKT7-MIF and pGADT7-candidate prey. DDO is Double Dropout:(SD/-Leu/-Trp) while DDO/X/A is DDO plus *X*-*α*-*gal* and *Aureobasidin*-A. QDO is quadruple dropout. **b** Full-length HTRA1 is a 480 amino acid protein containing an IGF binding region and a serine protease domain, between amino acids 204 and 364. In the extreme C-terminus a PDZ domain is found. The cDNA clone isolated by us (in the co-transformation) contained the coding sequence for the whole PDZ domain and part of the protease domain. To determine what part of the MIF molecule binds to HTRA1, we made C-terminally truncated constructs of MIF (*lines*). The truncations where made so that that the two α-helix’s where intact, since these are facing outwards in the MIF trimer. We also used software modeling to predict potential interaction sites on MIF and HTRA1 (**c**, **d**). Since there is no crystal structure for the HTRA1 molecule available containing both the protease domain and the PDZ domain, we modeled the two domains separately and both are predicted to interact with MIF (*pink*). The PDZ domain (*blue*) is predicted to interact with the loop between the N-terminal β-sheet and the first α-helix (**c**), while the protease domain (*green*) interacts with the first α-helix (**d**)

An yeast two-hybrid screen was performed using a human fetal brain cDNA library to identify proteins that bind to full-length MIF. Here we identified a new interacting protein: a member of the trypsin family of serine proteases, HTRA1. The cDNA clone isolated contained the coding sequence for the C-terminal 193 amino acids of HTRA1. Full-length HTRA1 contains 480 amino acids and the serine protease domain is found between amino acids 204 and 364. In the extreme C-terminus, following the protease domain, a PDZ domain is found. The cDNA clone we isolated therefore contained the coding sequence for the whole PDZ domain and part of the protease domain (Fig. 1a). The cDNA clone also contained non-coding sequences. To confirm that it was the HTRA1 coding sequences that resulted in a positive interaction in the yeast two-hybrid screen, all non-coding sequences were removed and the interaction was confirmed by direct mating in yeast.

To determine which part of the MIF molecule that is involved in the interaction with HTRA1, we made C-terminally truncated constructs of MIF (Fig. 1a). The truncations were made in a way that the two α-helixes were intact, since these are facing outwards in the MIF trimer. These truncated constructs were then used for direct mating experiments in yeast, showing that the first 38 amino acids of MIF, that contain the first α-helix, bind to HTRA1. We then made truncated HTRA1 constructs containing either the PDZ domain or the part of the protease domain that was pulled out of the cDNA library. When these constructs were used for direct mating experiments, no interaction was found. As this was an unexpected result, we used software modeling (Patchdock and FireDock) to investigate potential binding sites. Since there is no crystal structure for the HTRA1 molecule available containing both the protease domain and the PDZ domain, we modeled the two domains separately. The results suggest that both domains interact with MIF (Fig. 1b, c). The PDZ domain is predicted to interact with the loop between the N-terminal β-sheet and the first α-helix (Fig. 1b), while the protease domain interacts with the first α-helix (Fig. 1c). The modeling results are in agreement with the results from the yeast two-hybrid studies and suggest that both HTRA1 domains are needed to form a stable interaction with MIF.

### Verification of the binding between MIF and HTRA1

**Fig. 2:**
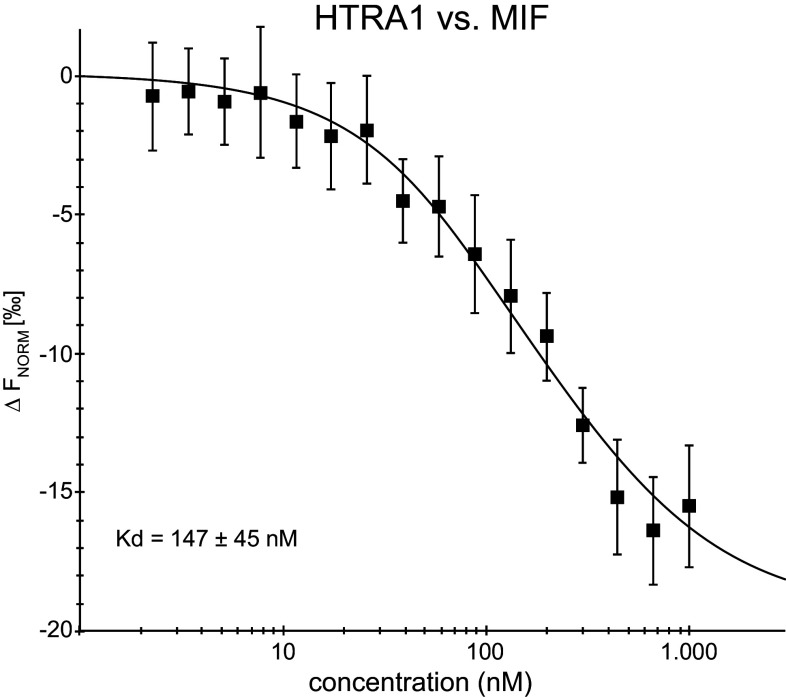
Interaction analysis of the MIF–HTRA1 binding: Microscale thermophoresis (MST) measurements were performed on the binding between HTRA1 and MIF. The MST measurements were made using a constant concentration of labeled HTRA1 while the concentration of the non-labeled MIF was varied. The MST measurements were performed using a Monolith NT.115. A *K*_D_ of 147 ± 16 nM was determined for this interaction employing the Thermophoresis + T-Jump signal for data analysis (*n* = 3 independent measurements, *error bars* represent the standard deviation)

The binding between MIF and HTRA1 was further verified using microscale thermophoresis (MST). The measurements were performed using a constant concentration of labeled HTRA1 (65 nM) while the concentration of the non-labeled MIF, varied between 2.2 nM–1 μM. A *K*_D_ of 147 ± 16 nM (*n* = 3) was determined for the interaction (Fig. 2). This *K*_D_ is in the physiological range and the binding is thus likely to occur in tissues such as the nervous system [58].

### MIF inhibits the enzymatic digestion mediated by HTRA1

**Fig. 3:**
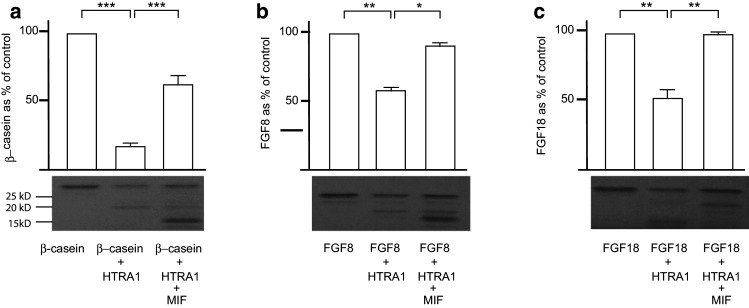
Functional analysis of the MIF–HTRA1 binding: the function of the MIF and HTRA1 binding was first tested with β-casein, a known substrate for HTRA1 (**a**). The addition of HTRA1 effectively cleaved the β-casein. A new sample was then mixed, adding β-casein together with HTRA1 and MIF. Interestingly, MIF effectively inhibited the HTRA1-mediated cleaving of β-casein. It is known that FGF8 is also cleaved by HTRA1. Therefore, the experiment was repeated with FGF8 instead of β-casein. HTRA1 cleaved FGF8 as previously described, but the addition of MIF effectively inhibited the cleavage (**b**). FGF18, which is closely related to FGF8, was also effectively cleaved by HTRA1 and the cleavage was significantly inhibited by MIF (**c**). The coomassie gel for each experiment is added *below* the diagram. *Error bars* represent SEM, *n* = 3. *p  <  0.05, **p  <  0.01, ***p < 0.001

We next tested the function of the MIF–HTRA1 interaction. HTRA1 has been reported to cleave β-casein [13] and we therefore replicated this in our experiments. Interestingly, the addition of MIF to HTRA1 before the addition of β-casein, inhibited the HTRA1-mediated enzymatic breakdown significantly (Fig. 3a). HTRA1 is also known to enzymatically digest fibroblast growth factor-8 (FGF8) [13]. Next, we investigated if MIF had the ability to inhibit the enzymatic digestion of this protein as well, and this was indeed the case (Fig. 3b). There are two other members of the FGF family, FGF17 and FGF18 that share a high sequence homology with FGF8 [2]. We tested if HTRA1 had the ability to enzymatically break down these proteins. It was found that HTRA1 could enzymatically digest FGF18, but not FGF17 and that MIF could inhibit the enzymatic breakdown of this protein too (Fig. 3c).

### MIF and HTRA1 expression in vitro

**Fig. 4:**
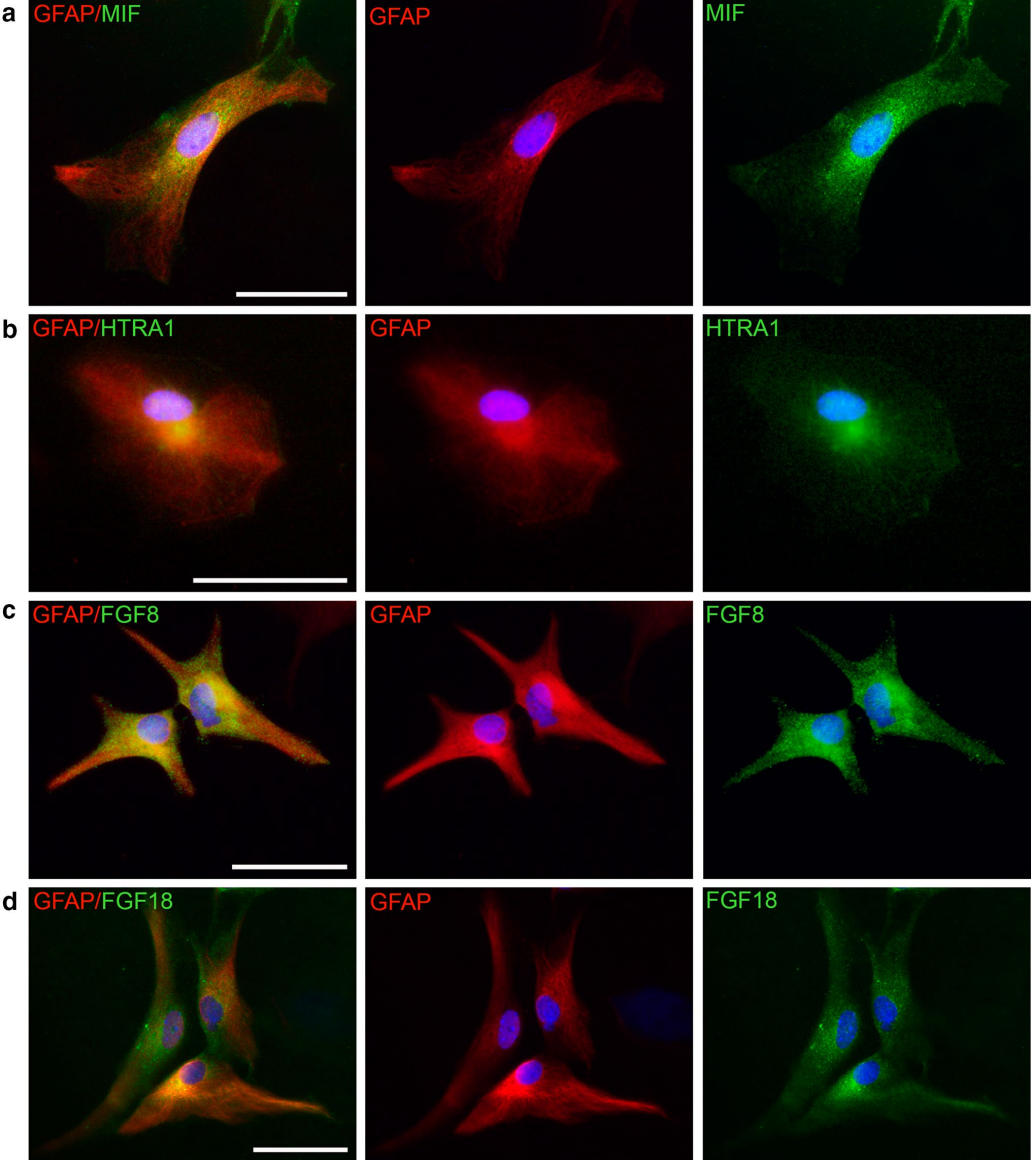
Immunochemical investigation of protein expression: The presence of MIF (**a**), HTRA1 (**b**), FGF8 (**c**) and FGF18 (**d**) were investigated in cultured mouse astrocytes co-labeled with GFAP. It was found that the astrocytes express MIF as well as HTRA1 in their cytoplasm. The somewhat grainy labeling of MIF may indicate that the protein is stored in vesicles in the cytoplasm. HTRA1 is also localized to the cytoplasm, as are FGF8 and FGF18. DAPI was used for nuclear staining. *Error bars* are 50 µm

The distribution of MIF in the CNS has previously been studied and MIF is present in most cells of the CNS [12, 59–61]. However, the distribution of HTRA1 in the CNS is relatively unknown although, as mentioned previously, it has been found in both neurons as well as glia [33, 36]. We have previously found that MIF is highly expressed in astrocytes [62] and that this cell type express HTRA1 as well. Unfortunately, we could not obtain data of complete co-localization since the working antibodies are both made within the rabbit immune system. As shown in Fig. 4, astrocytes labeled with GFAP express both MIF and HTRA1 (Fig. 4a, b), and the proteins have a similar distribution. Since HTRA1 enzymatically digests both FGF8 and FGF18, we also chose to determine if these proteins were present in astrocytes. It was found that astrocytes do express FGF8 as well as FGF18 (Fig. 4c, d).

### HTRA1 decreases FGF8-induced astrocyte migration, while MIF inhibits this HTRA1-mediated effect

**Fig. 5:**
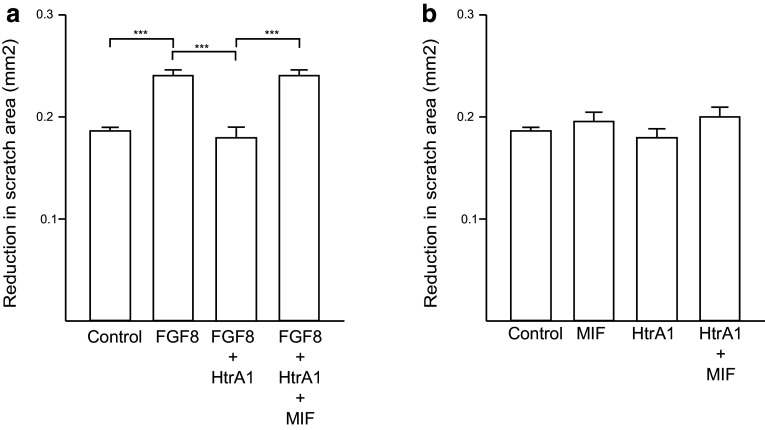
In vitro functional analysis using cultured mouse astrocytes: MIF blocks HTRA1-mediated inhibition of FGF8-stimulated astrocyte migration (**a**). Using the scratch assay, astrocyte migration was enhanced using FGF8. When HTRA1 was added in combination with FGF8, the migration decreased significantly. In cultures where FGF8, HTRA1 and MIF were added together, astrocyte migration significantly increased to a level similar to that of cultures that only received FGF8, *n* = 5. HTRA1 or MIF in combination or alone added to astrocyte cultures did not have any effect on migration in the cultures (**b**), *n* = 3. The same experiment was also made with FGF18, with the same result. *Error bars* represent SEM. *p  <  0.05, **p  <  0.01, ***p < 0.001

Since both MIF and HTRA1 are present in astrocytes, we went on to study the function of the MIF–HTRA1 interaction in this cell type. It has previously been shown that FGF8 influences the migration, but not proliferation of astrocytes in vitro using a scratch/wound healing assay [43, 54, 63]. Since FGF8 mediates increased migration, we reasoned that this effect could be inhibited by HTRA1. Replicating Kang et al. [43], astrocyte migration was first investigated with and without FGF8. In cultures where FGF8 was added, astrocytes migration was significantly increased compared to control cultures (Fig. 5a), whereas cell proliferation was not affected (data not shown). When HTRA1 was added in combination with FGF8, the migration decreased significantly (Fig. 5a). In fact, the migration in these cultures was similar to the migration levels in the control cultures. In cultures where FGF8, HTRA1 and MIF were added together, astrocyte migration significantly increased to a level similar to that of cultures that only received FGF8 (Fig. 5a). HTRA1 or MIF in combination or alone, added to astrocyte cultures, did not have any effect on migration in the cultures (Fig. 5b).

## Discussion

MIF is highly expressed throughout the central nervous system (CNS) and identified in most cells in nervous tissue including neurons, ependymal cells, astrocytes, oligodendrocytes, neural stem cells/progenitor cells, activated microglia, and Schwann cells [59, 62, 64, 65]. Several previous investigations have suggested that MIF may be involved in nervous system development, though the function of MIF in this setting is far from clear, and receptor as well as binding partner distributions are unknown [4, 12, 66]. To delineate this, we searched for new MIF binding partners in the CNS using a human fetal brain cDNA library and found HTRA1, an enzyme that belongs to a family of four members (the serine proteases 1–4). These family members share a relatively high sequence homology, but differ in cellular functions [32]. The primary structure of the protein consists of five different parts: signal peptide (SP), insulin-like growth factor-binding domain (IGF-BP), Kazal inhibitor (KI), protease, and PDZ domain. In the case of the MIF–HTRA1 binding, we found that MIF likely binds to both the PDZ domain and the protease domain. The yeast hybrid and protein modeling results (Fig. 1b, c) also suggest that stability in this binding is only accomplished by binding to both domains.

Several proteolytic substrates for HTRA1 have been identified, as for example insulin-like growth factor-binding protein 5 (IGF-BP-5), fibroblast growth factor-8 (FGF8), transforming growth factor β (TGF- β) as well as extracellular matrix components such as aggrecan, decorin, fibromodulin and soluble type II collagen [13, 35, 36, 40, 42, 67].

Since HTRA1 enzymatically digest FGF8 [13], we tested if MIF binding to HTRA1 would inhibit this enzymatic breakdown. Interestingly, this was the case. We also tested if HTRA1 would break down the close relatives to FGF8, FGF17 and FGF18. Surprisingly, we found that HTRA1 digested FGF18, but not FGF17, although the breakdown of FGF18 needed a higher concentration of HTRA1 and a longer incubation period than for FGF8. MIF, in turn, also had the ability to inhibit the breakdown of both FGF8 and FGF18. Since both MIF and HTRA1 expression are common in most tissues, we suggest that the inhibition of HTRA1-mediated enzymatic breakdown by MIF is commonly occurring in tissues where both proteins are present. No endogenous proteins that have the ability to regulate HTRA1’s protease function have been found previously [68]. Thus, MIF is the first to do so.

As stated previously, both MIF and HTRA1 are present in most cells of the CNS. We found that astrocytes express MIF and HTRA1 as well as FGF8 and FGF18. Young astrocytes have a relatively higher immunoreactivity for all of the proteins mentioned (data not shown). This is also in accord with previous findings showing that HTRA1, FGF8 as well as FGF18 are implicated in brain development [36, 69, 70].

To investigate the function of the MIF–HTRA1 binding, we used cultured mouse astrocytes. It has previously been shown that FGF8 increases migration, but not proliferation in such cells [43]. We investigated if HTRA1 would inhibit this migration and indeed this was the case. Adding FGF8, HTRA1 and MIF together to the cells, the migration was restored. Neither MIF nor HTRA1 affect astrocyte migration on its own. MIF is stored in intracellular pools, and several hormonal, mitogenic, and pro-inflammatory stimuli can elicit its secretion [71]. Although several secretion-stimulating factors such as glucocorticoids, INFγ, LPS and hypoxia have been identified, the exact mechanism of secretion is not known [72, 73]. Both HTRA1 and FGF8 are secreted from cells during development and in adult organisms [42, 74]. Since astrocytes express all three proteins, it is conceivable that MIF and HTRA1 interact during certain conditions in the CNS. Our data suggest that MIF may aid regeneration after CNS injury, by inhibiting HTRA1 that is continuously secreted from cells. An increase of MIF thus contributes to the increase of growth factors, such as FGF8, FGF18 and TGF-β that in turn can increase astrocyte and oligodendrocyte migration and proliferation as well as neural protection [75–77].

### Electronic supplementary material

Below is the link to the electronic supplementary material.

**Supplementary Figure** **1** The activity of the HTRA1 transfected HEK293 cell media was tested on β-casein. It was also investigated if media from normal untransfected HEK293 cells could cleave the β-casein but this was not the case (A). The minimum amount of HTRA1 media that could cleave 1 µg β-casein was then tested. In this test a cleavage could be seen down to 4 µL of HTRA1 containing HEK293 media (B). The amount of MIF needed to inhibit HTRA1 cleaving was next tested. Here it was found that the lowest amount of MIF that could stop the HTRA1 medium from cleaving 1 µg β-casein was 1 µg MIF (C). (PDF 1664 kb)
