# Supplementary figures and images for: Macrophage migration inhibitory factor (MIF) modulates trophic signaling through interaction with serine protease HTRA1
Source: Cell Mol Life Sci. 2017 Jul 19;74(24):4561–72. doi: 10.1007/s00018-017-2592-z (PMC5663815; doi:10.1007/s00018-017-2592-z)

A.

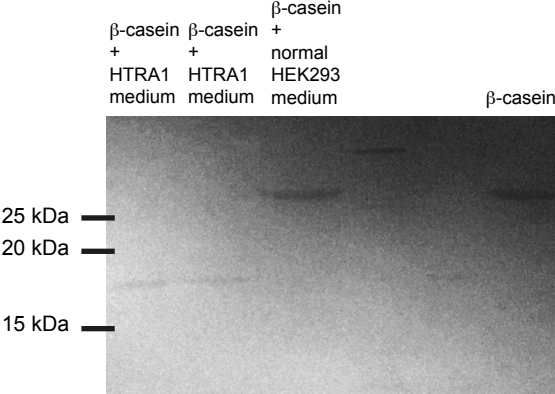

B.

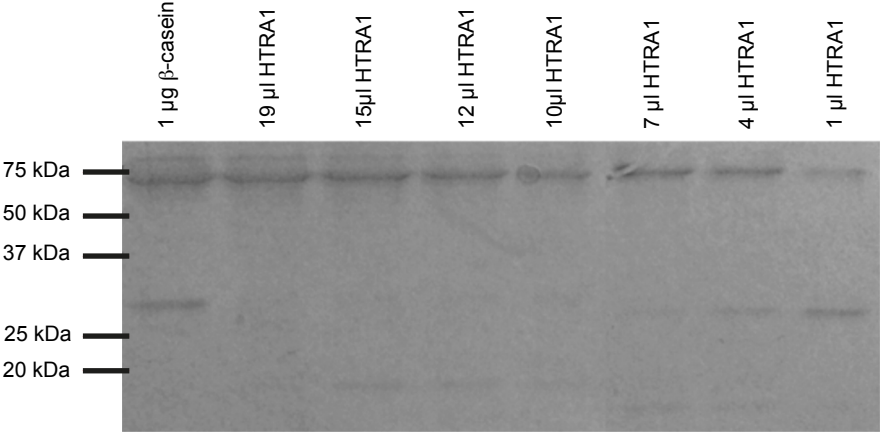

C.

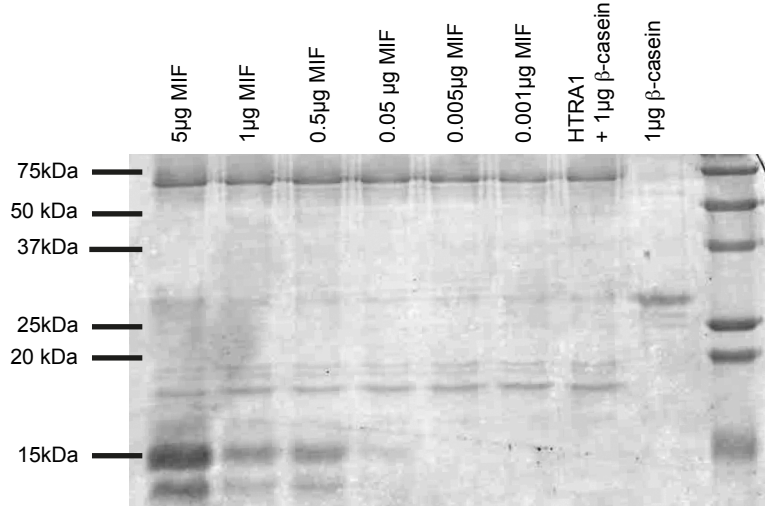

Supplement: Supplementary file 1 — Supplementary Figure 1 The activity of the HTRA1 transfected HEK293 cell media was tested on β-casein. It was also investigated if media from normal untransfected HEK293 cells could cleave the β-casein but this was not the case (A). The minimum amount of HTRA1 media that could cleave 1 µg β-casein was then tested. In this test a cleavage could be seen down to 4 µL of HTRA1 containing HEK293 media (B). The amount of MIF needed to inhibit HTRA1 cleaving was next tested. Here it was found that the lowest amount of MIF that could stop the HTRA1 medium from cleaving 1 µg β-casein was 1 µg MIF (C). (PDF 1664 kb) [file 18_2017_2592_MOESM1_ESM.pdf]
